# P-2204. Design and Pilot Implementation of an ECHO Module to Facilitate Treatment of Hepatitis C Virus Infection During Pregnancy

**DOI:** 10.1093/ofid/ofae631.2358

**Published:** 2025-01-29

**Authors:** Lynn Yee, Andrew Aronsohn, Seema Shah, Karen Lee, Isa Rodriguez, Sebastian Otero, Patrick Gower, Joseph D Fishbein, Daniel Johnson, Ravi Jhaveri

**Affiliations:** Northwestern University Feinberg School of Medicine, Chicago, Illinois; Univeristy of Chicago Hospitals, Chicago, Illinois; Ann & Robert H. Lurie Children's Hospital of Chicago, Chicago, Illinois; ECHO Chicago, Chicago, Illinois; ECHO Chicago, Chicago, Illinois; ECHO Chicago, Chicago, Illinois; ECHO Chicago, Chicago, Illinois; Ann and Robert H. Lurie Children's Hospital of Chicago, Hinsdale, Illinois; Univeristy of Chicago Hospitals, Chicago, Illinois; Ann & Robert H. Lurie Children's Hospital of Chicago, Chicago, Illinois

## Abstract

**Background:**

Pregnant patients are largely excluded from Hepatitis C virus (HCV) research and treatment. Studies show that pregnant patients want to discuss treatment while providers feel they lack the knowledge and training for these discussions. To bridge this gap, we designed an ECHO module about management of HCV in pregnancy and infancy.

Pre- and Post-surveys of Self Efficacy

**Figure:**
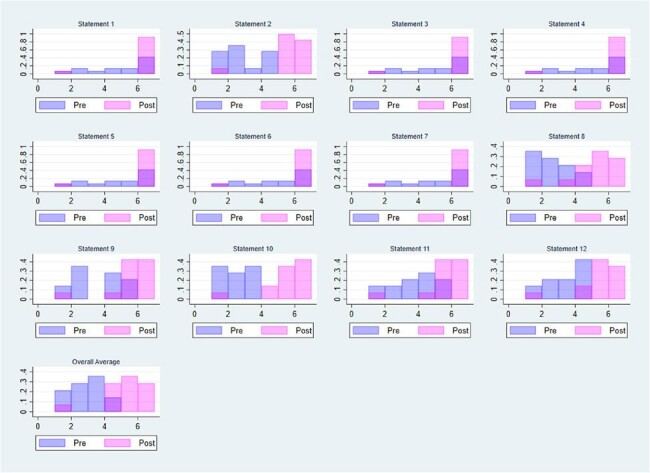

Increase in self-efficacy scores after participation in the ECHO module about HCV care during pregnancy

**Methods:**

Content experts from pediatric infectious diseases, hepatology, maternal-fetal medicine, bioethics and members of ECHO-Chicago discussed: prior data, potential topics, target audience and scheduling of sessions. We held 8 one-hour sessions with 20-30 minutes of didactics and 30-40 minutes of solicited case discussion. Participants included those who participated in prior ECHO training and prenatal providers in local and regional practices. A 7-point Likert scale pre- and post-training survey was administered to assess metrics of self-efficacy for management of HCV in pregnancy and childhood.

**Results:**

The sessions were conducted from May-June 2023 on the following topics: HCV screening; HCV pathophysiology/clinical presentations; staging fibrosis in HCV; HCV Treatment in non-pregnant patients; harm reduction; clinical considerations of HCV in pregnancy; shared decision-making for HCV treatment in pregnancy; and HCV screening in infants & treatment for children. Fourteen participants completed pre- and post-surveys. In the pre-survey, participants had low ratings of self-efficacy (average 3.0). In the post-survey, participants reported dramatic increases in all areas (average 5.2, increase of 2.2) with the largest in the following areas: “Ability to discuss and manage timing of hepatitis C treatment in a pregnant person”, average 5.1, increase of 3; “Ability to identify suitable pregnant candidates for treatment for Hepatitis C”, average 5.2, increase of 2.7. For context, a 1-point increase in self-efficacy is considered meaningful. All respondents reported increased ability to address HCV in a preconception, pregnant, postpartum, or neonatal population.

**Conclusion:**

As pivotal studies of pan-genotypic antivirals indicate efficacy and safety during pregnancy, this ECHO module could be distributed to support providers who are discussing therapy initiation.

**Disclosures:**

Ravi Jhaveri, MD, AstraZeneca: Advisor/Consultant|Gilead: Advisor/Consultant|GSK: Grant/Research Support|Pediatric Infectious Diseases Society: editorial stipend|Sanofi: Advisor/Consultant|Seqirus: Advisor/Consultant|UpToDate: royalties

